# Humoral immunity after hematopoietic stem cell transplantation: evaluation by B-cell receptor repertoire analysis

**DOI:** 10.1007/s12185-025-04042-9

**Published:** 2025-07-25

**Authors:** Sakuya Matsumoto, Yohei Funakoshi, Kimikazu Yakushijin, Takaji Matsutani, Yuri Okazoe-Hirakawa, Goh Ohji, Taiji Koyama, Yoshiaki Nagatani, Keiji Kurata, Shiro Kimbara, Naomi Kiyota, Hironobu Minami

**Affiliations:** 1 Division of Medical Oncology/Hematology, Department of Medicine, Kobe University Hospital and Graduate School of Medicine, Kobe, Japan; 2 Translational Research Dept. Maruho Co., Ltd., Kyoto, Japan; 3 Division of Infection Disease Therapeutics, Department of Microbiology and Infectious Diseases, Kobe University Hospital and Graduate School of Medicine, Kobe, Japan; 4 Cancer Center, Kobe University Hospital, Kobe, Japan

**Keywords:** Hematopoietic stem cell transplantation, mRNA vaccine, B-cell receptor repertoire, Coronavirus Antibody Database, Quantification of antigen-specific antibody sequence

## Abstract

Immunity acquired before hematopoietic stem cell transplantation (HSCT) may decrease or disappear following HSCT, and it is unclear whether the first vaccination after HSCT in patients with an antigen exposure history before HSCT elicits a primary or secondary immune response. The Quantification of Antigen-Specific Antibody Sequence (QASAS) method enables real-time assessment of responses to SARS-CoV-2 antigen exposure through B-cell receptor (BCR) repertoire analysis. Using this method, we evaluated the disappearance of immunological memory after HSCT. First, in individuals without hematologic disorders, primary SARS-CoV-2 antigen exposure elicited no immune response at 7 days post-exposure but demonstrated activation between 14 to 21 days. In contrast, repeated exposure elicited early responses (secondary immune responses) at 7 days post-exposure. We then enrolled HSCT patients with pre-HSCT SARS-CoV-2 antigen exposure history and collected samples before and after vaccination. Despite prior exposure history, patients receiving their first vaccination after HSCT showed no response around 7 days post-exposure but responded at 14 days. In conclusion, even with pre-HSCT antigen exposure, the first vaccination after HSCT induced a primary immune response. This demonstrates that first vaccination after HSCT should be considered to induce a primary immune response, regardless of previous infection or vaccination history.

**Supplementary Information:**

The online version contains supplementary material available at 10.1007/s12185-025-04042-9.

## Introduction

Hematopoietic stem cell transplantation (HSCT) is a therapeutic approach that utilizes hematopoietic stem cells to cure a variety of hematological diseases. These include acute and chronic leukemia, lymphoma, certain inherited disorders of the hematopoietic system, and metabolic conditions [1, 2]. During HSCT, the recipient's immune system is replaced with an immune system derived from donor hematopoietic stem cells via allografting [3, 4]. Consequently, immunity acquired through vaccination or natural infection prior to HSCT is believed to be significantly diminished or entirely lost. In the absence of reimmunization, immunity against many pathogens declines following HSCT. Accordingly, prophylactic or pre-emptive strategies to prevent infection in these patients are recommended, including the post-HSCT use of antibiotics, antivirals, antifungals, and revaccination. Notably, guidelines strongly advocate revaccination against vaccine-preventable diseases, such as influenza, pneumococcal infections, rubella, and measles [5, 6]. mRNA vaccines introduced against SARS-CoV-2 in 2021 have demonstrated good immunogenicity even in HSCT recipients, as we previously reported, and revaccination after HSCT against COVID-19 is also recommended [7, 8].

Although revaccination after HSCT is therefore necessary, the change in immune status following HSCT remains inadequately characterized. A major uncertainty is whether the immune memory is entirely erased following HSCT or partially preserved. This ambiguity complicates decision making regarding the optimal number of vaccine doses required post-HSCT. Furthermore, although vaccine efficacy is considered closely linked to immune status at the time of vaccination, the status of reconstructed acquired immunity, especially humoral immunity, after HSCT remains unclear. Previous studies have indicated that B-cell counts may recover within a few months following HSCT [9]; however, many of these cells remain immature, potentially resulting in differences in the function and diversity (repertoire) of humoral immunity compared to healthy individuals [10, 11]. We therefore considered that optimizing revaccination strategies after HSCT, including appropriate timing and methods, required a detailed understanding of the immune status following HSCT.

We previously established a novel method to assess the response to SARS-CoV-2 vaccination at the mRNA level by quantifying antigen-specific antibody sequences, named the “Quantification of Antigen-specific Antibody Sequence (QASAS) method” [12–14]. Compared to conventional tests, which detect antibodies with a long half‐life, the QASAS method directly detects B-cell receptor (BCR) mRNA as short‐lived, allowing humoral immunological activity to be monitored in real time [12–14]. We hypothesized that use of the QASAS method to analyze immune responses in detail following vaccination in post-transplant patients would allow us to assess immune status after HSCT.

Although the impact of HSCT on the immune system has been extensively studied, studies have primarily focused on T cells, and information on its effects on B-cell/humoral immunity is limited. Furthermore, while the genetic diversity of BCRs is a crucial indicator of humoral immune status, its alteration following HSCT has not been fully elucidated.

Here, to support the development of strategies to prevent post-transplant infection, we evaluated the loss of immunological memory and the status of humoral immunity after HSCT using various analytical approaches. We particularly focused on BCR repertoire analysis.

## Materials and methods

### Participants

For flow cytometry (fluorescence‑activated cell sorting; FACS) analysis, 10 HSCT patients (patients 1 to 10; cord blood, *n* = 3; bone marrow, *n* = 7) were enrolled (Table S1). To investigate humoral responses to SARS‑CoV‑2 using the QASAS method, we collected 14 datasets (cases 1 to 14) of SARS-CoV-2 antigen exposure from nine individuals without hematologic disorders (Table S2). Then, to examine vaccine‑induced immunity after HSCT, we further enrolled three HSCT patients (patients 11–13) who received cord blood transplantation with a history of exposure to SARS-CoV-2 antigen before HSCT (Table S3).

The study protocol was approved by the Kobe University Hospital Ethics Committee (Protocol nos. B2056704 and 1481) and conducted in accordance with the Declaration of Helsinki. All samples were collected at Kobe University Hospital between July 2020 and January 2024. Written informed consent was obtained from all participants.

### Sample collection and processing

Peripheral blood samples were collected using heparin-containing tubes. Peripheral blood mononuclear cells (PBMCs) were isolated from the blood by density gradient centrifugation using Lymphoprep (Serumwerk Bernburg AG, Bernburg, Germany) and SepMate-50 tubes (STEMCELL Technologies Inc., Vancouver, BC, Canada). PBMC samples were stored with CELLBANKER (Zenogen Pharma, Fukushima, Japan) at − 80 °C until analysis. Total RNA was extracted with TRIzol LS (Thermo Fisher Scientific, Waltham, MA, USA) and purified with an RNeasy Mini Kit (Qiagen, Hilden, Germany).

### Flow cytometric analysis

PBMC were stained for 60 min at 4 °C with anti‑human antibodies for the B-cell lineage, namely CD19 BV510 (Biolegend, San Diego, CA, USA), CD27 BV421 (Biolegend), IgD FITC (Biolegend), CD24 PerCP-Cy^TM^5.5 (BD Biosciences, Franklin Lakes, NJ, USA) and CD38 BV711 (Biolegend). Isotype-matched antibodies served as controls. Flow cytometric analysis was performed using a FACSAria III instrument (BD Biosciences) with established phenotypes used for B-cell analysis. CD19^+^ cells were defined as total B cells. The following subsets of B cells were then analyzed: transitional B cells (CD19^+^ CD24^+^ CD38^+^) and class-switched B cells (CD19^+^ CD27^+^ IgD^−^).

### B-cell receptor repertoire analysis

BCR repertoire analysis was performed according to previous studies [12–14]. For mutation analysis, paired-end reads were assembled into a complete sequence using the AssemblePairs.py command of the pRESTO package (https://presto.readthedocs.io/en/stable/). The fasta-formatted assembled sequence was analyzed for immunoglobulin gene rearrangement using igblast-1.22.0 and output as adaptive immune receptor repertoire (AIRR)-formatted data. Sequence identity to the IMGT reference sequence (v_identity and j_identity) was used for mutation analysis. Probability of the generation (pGen) of amino acid sequences of CDR3 was computed from a generative model of V(D)J recombination using the OLGA packages [15]. CDR3 sequences that failed to compute pGen were excluded from subsequent analysis.

### Database and COVID-19-specific sequence search

Antibody sequences specific to COVID-19 were downloaded from CoV-AbDab (http://opig.stats.ox.ac.uk/webapps/covabdab/). Data which had been updated on 20 December 2022 with 12,004 entries were used as reference. A total of 260,856,092 sequences were used to validate the method. IgG and IgM antibody sequences from 12 healthy volunteers before the COVID-19 pandemic were described previously [16]. Sequences with V and J gene names identical to the query sequence and with CDR3 amino acid sequence differences (Levenshtein distance) of 0 to 2 were retrieved from the database.

### Statistical analyses

Shannon–Weaver diversity index was calculated with the vegan package in R and tested for significant differences using the Wilcoxon test. Levels of somatic hypermutation between HSCT patients and healthy controls were compared with the Wilcoxon test. All *p* values were two sided and considered statistically significant at the < 0.05 level. Statistical analyses were carried out with GraphPad Prism 10.0 (GraphPad Software, San Diego, CA, USA).

## Results

### Transition of B-cell subsets after HSCT

**Fig. 1:**
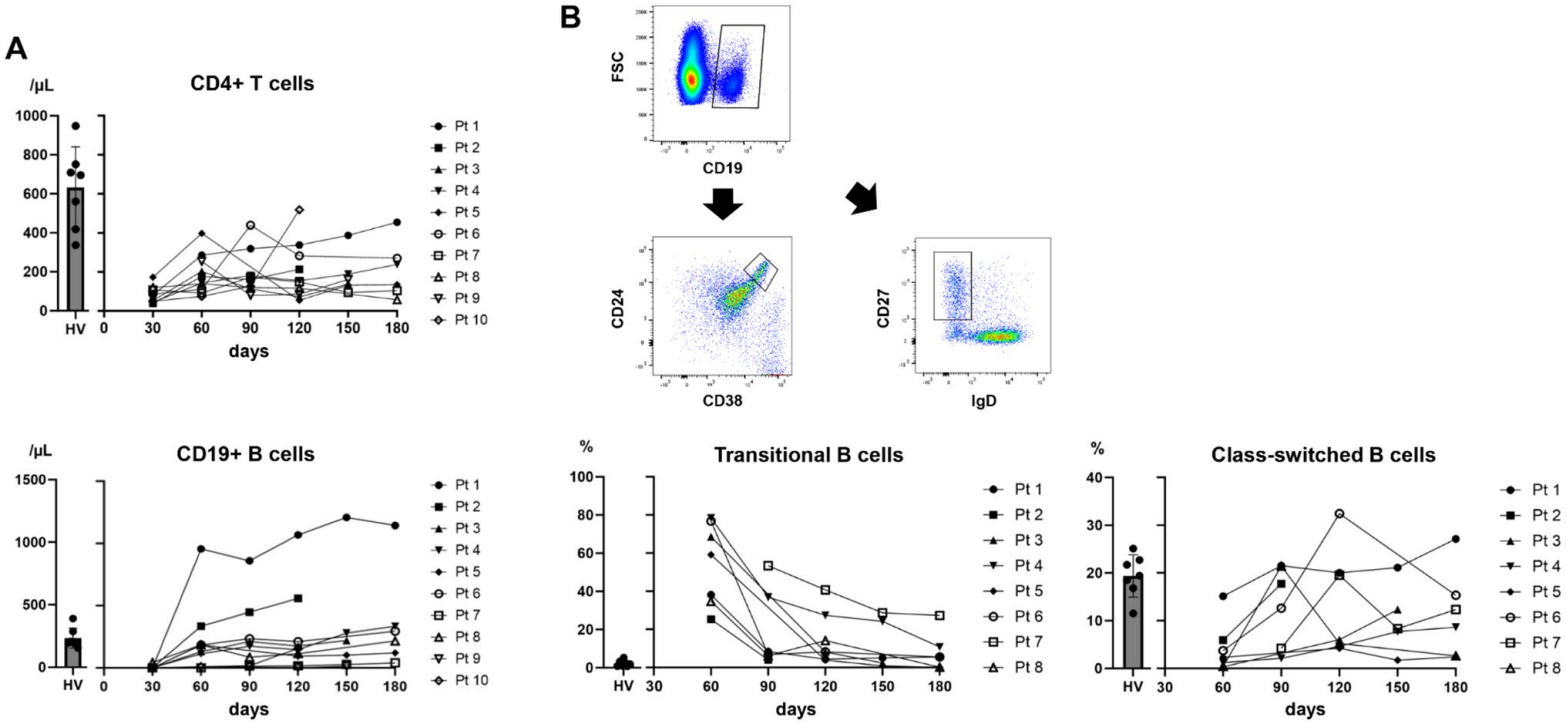
B-cell reconstitution after HSCT: **A** PBMCs were collected from patients at various time points after HSCT and CD4^+^ T cells and CD19^+^ B cell counts were analyzed by FACS. HV, healthy volunteers. **B** Gating strategies used to identify transitional and class-switched B cells based on CD24/CD38 and CD27/IgD expression profiles within the CD19 ^+^ B-cell population. The percentage of transitional and class-switched B cells was chronologically analyzed after HSCT by FACS. HV, healthy volunteers

To assessed the status of lymphocyte subpopulations in patients who had undergone HSCT using FACS, peripheral blood samples were serially collected from 10 HSCT patients (patients 1 to 10) (cord blood, *n* = 3; bone marrow, *n* = 7) and their lymphocyte subpopulations (CD4⁺ T cells and CD19⁺ B cells) were quantified by FACS (Table S1). Details of the HSCT patients are shown in Table S1. CD4^+^ T cells were observed to increase from approximately day 30 after HSCT (Fig. 1A). In contrast, B cells were rarely detectable in peripheral blood at day 30 after HSCT but began to appear around day 60, with levels exceeding normal ranges in some patients (Fig. 1A).

To further evaluate B-cell recovery, we analyzed transitional and class-switched B cells using CD24/CD38 and CD27/IgD expression profiles on CD19^+^ B cells (Fig. 1B). At day 60, when B cells first reappeared in peripheral blood, the majority consisted of immature transitional B cells, whereas the proportion of more mature class-switched B cells remained low (Fig. 1B). These findings suggested that, during the early phase of immune reconstitution following HSCT, mature B cells are still largely lost, and there is a period in which the B-cell compartment is predominantly composed of immature cells which are differentiated from hematopoietic stem cells.

### Reset of acquired immunity through HSCT

**Fig. 2:**
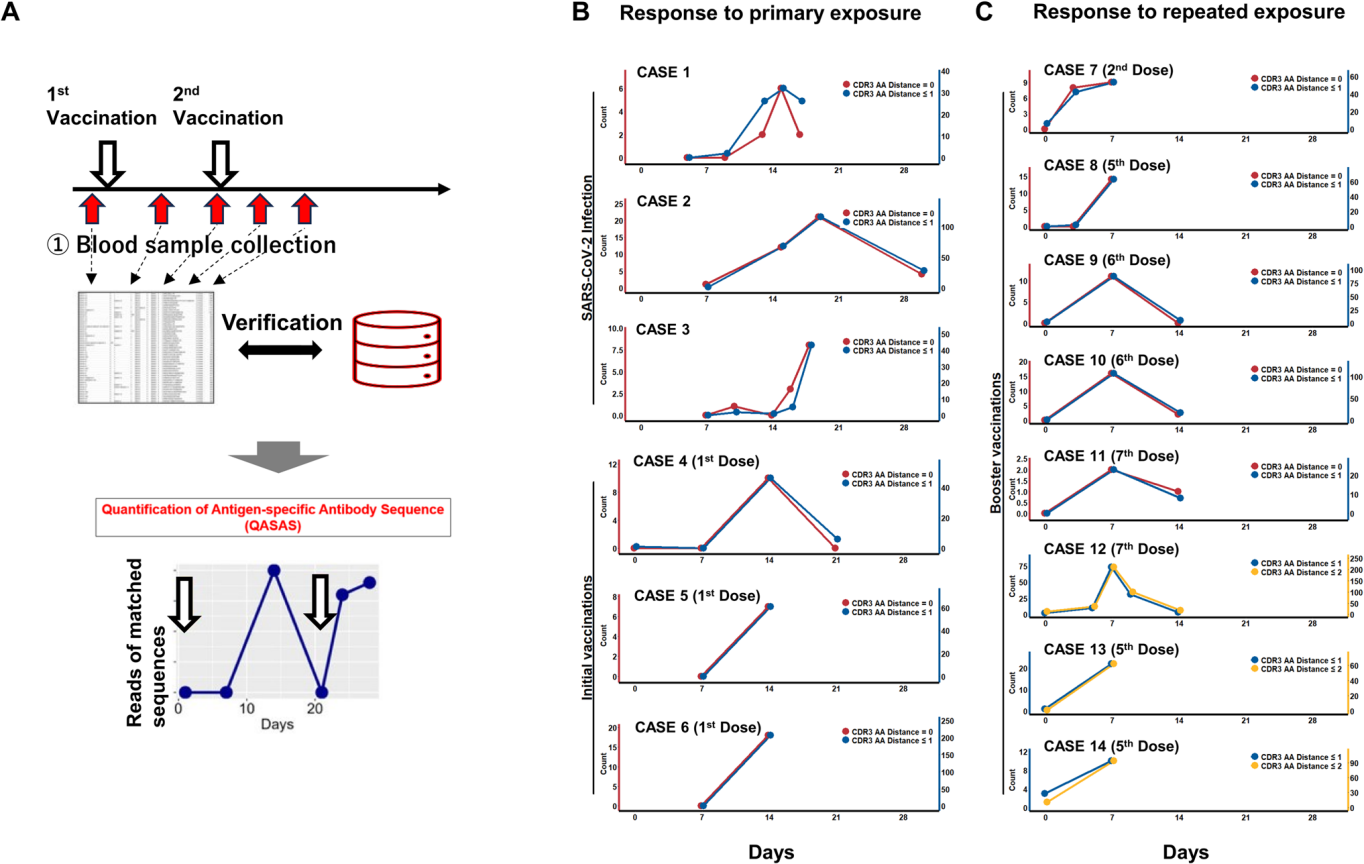
Schema of the QASAS method: **A** We collected blood samples over time pre- and post-vaccination to analyze the BCR repertoire. We then analyze the extent to which BCR sequences in the database were contained in the obtained BCR sequence data set, and analyzed the transition of matched BCR sequences. Immune activation timing between primary and repeated exposure by the QASAS method: **B**, **C** Changes over time in the number of SARS-CoV-2-specific sequences following SARS-CoV-2 antigen exposure. Before and after exposure, SARS-CoV-2-specific sequences were retrieved from the BCR repertoire data. We evaluated immune responses using the QASAS method in six cases with primary exposure to SARS-CoV-2 antigen (infections, *n* = 3; vaccinations, *n* = 3) and in eight cases with repeated exposure (vaccinations, *n* = 8) from nine individuals without hematologic disorders

We hypothesized that immune status could be assessed by analyzing immune activation following vaccination using the QASAS method (Fig. 2A). To investigate this possibility, we collected 14 datasets (cases 1–14) with SARS-CoV-2 antigen exposure from nine individuals without hematologic disorders (Fig. 2B, C and Table S2). We evaluated immune responses using the QASAS method in six cases with primary exposure to SARS-CoV-2 antigen (infections, *n* = 3; vaccinations, *n* = 3) (Fig. 2B and Table S2) and in eight cases with repeated exposure (vaccinations, *n* = 8) (Fig. 2C and Table S2). Cases with primary exposure showed no response at 7 days post-exposure but did show activation at between 14 and 21 days (Fig. 2B). In contrast, all cases with repeated exposure showed early responses (secondary immune responses) within 7 days (Fig. 2C). Since the timing of immune activation after primary exposure and repeated exposure was clearly different, it is possible to determine whether the immune response functions as a primary or secondary response using the QASAS method.

**Fig. 3:**
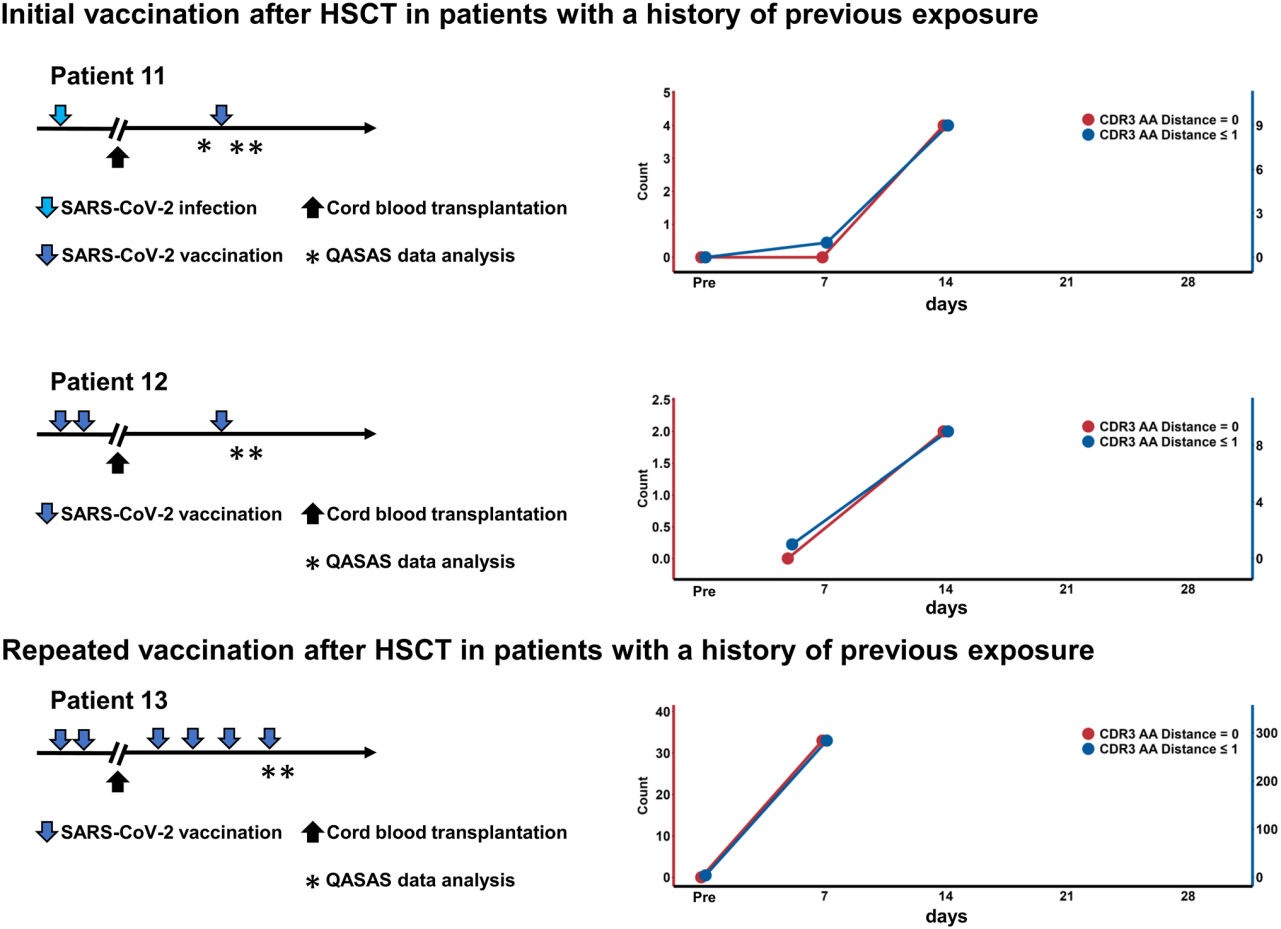
Immune activation timing in HSCT patients by the QASAS method: Three HSCT patients with a history of exposure to SARS-CoV-2 antigen before HSCT were enrolled. We evaluated immune responses in two patients at the time of initial vaccination after HSCT (patients 11 and 12) and in one patient with repeated vaccinations after HSCT (patient 13). SARS-CoV-2-specific sequences were measured by the QASAS method before and after vaccination

Next, to understand the immune status after HSCT, we evaluated the immune response to the SARS-CoV-2 vaccine after HSCT using the QASAS method. We further enrolled three HSCT patients (patients 11–13) who received cord blood transplantation with a history of exposure to SARS-CoV-2 antigen before HSCT (Table S3). We evaluated immune responses in two patients (patients 11 and 12) at the time of first vaccination after HSCT and in one patient with repeated vaccinations after HSCT (patient 13) (Fig. 3). Vaccination was administered at 420, 392, and 656 days following HSCT in patients 11, 12, and 13, respectively. Interestingly, despite a prior exposure history, the patients with first vaccination after HSCT showed no response around 7 days post-exposure, but instead at 14 days (Fig. 3). In contrast, the patient with repeated vaccinations after HSCT showed a response at 7 days post-exposure (Fig. 3). These findings suggested that acquired immunity was reset following HSCT.

### Evaluation of B-cell maturation after HSCT by BCR-repertoire analysis

**Fig. 4:**
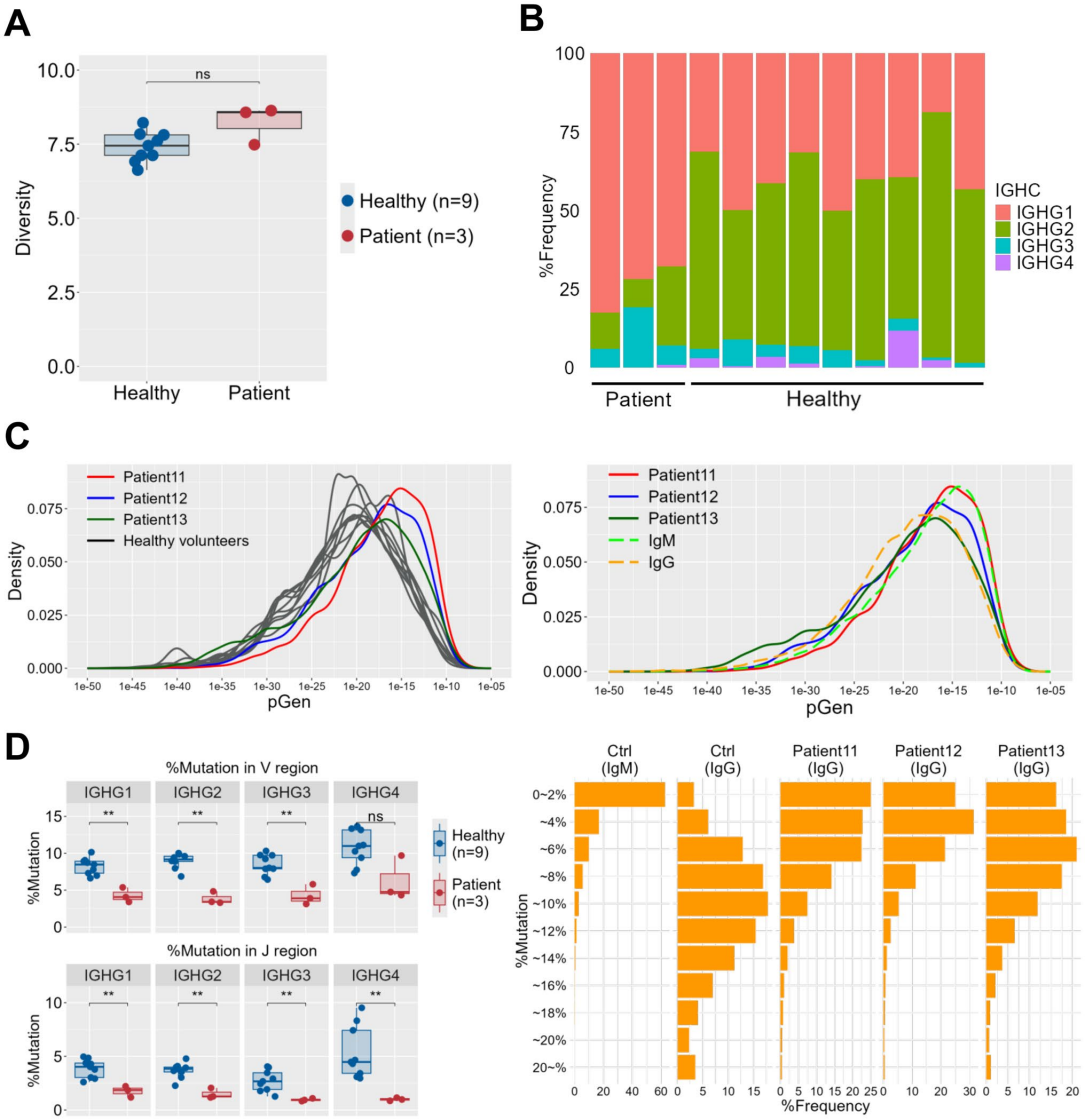
**A** Diversity of BCR repertoire in cord blood transplant patients and healthy controls. **B** Frequencies of IgG subclasses between HSCT patients and healthy controls: IgG subclasses (IgG1:IGHG1, IgG2:IGHG2, IgG3:IGHG3 and IgG4:IGHG4) were identified by the C region sequence of the BCR. Percentage frequencies of the read abundancies of each subclass are shown by a stacked bar graph. **C** Density plots of pGen values in patients and healthy controls (left). The density distribution of pGen values for three patients and nine healthy subjects shown in color (red: Patient 11, blue: Patient 12, green: Patient 13) and black, respectively. Distribution of pGen values between patients and control IgM and IgG subclasses (right). IgM and IgG sequences in pre-pandemic healthy adults were used as controls. **D** Levels of somatic hypermutation between HSCT patients and healthy controls (left). Percentage frequencies of mutation in nucleotide acid sequence in respective IgG subclasses were calculated for the V (upper) and J regions (lower). Distribution of somatic hypermutation levels in patients and control IgM and IgG subclasses (right). Histograms of the percentage of BCR sequences with different levels of mutation in the V region. IgM and IgG sequences in healthy adults were used as controls

To characterize humoral immunity in cord blood transplant patients, we analyzed the pre-vaccination BCR repertoire of three HSCT patients (patients 11 to 13) and nine healthy adults (controls). Comparison of the Shannon–Weaver diversity index showed no significant difference between the two groups (Fig. 4A). Using the constant (C) region of each sequence, we then assigned IgG subclasses and compared their frequencies. HSCT patients displayed a higher proportion of IgG1 and a lower proportion of IgG2 than healthy controls (Fig. 4B).

The probability of generation (pGen) estimates how likely a particular BCR sequence is to arise through V(D)J recombination. Antigen-naïve BCRs typically exhibit higher pGen values than antigen-experienced BCRs [17]. In the three HSCT patients (red, blue, and green lines) (Fig. 4C, left), the pGen distribution of IgG sequences was shifted to higher values relative to nine healthy controls (black lines) (Fig. 4C, left) and approximated the pGen values of IgM in nine healthy controls (Fig. 4C, right).

Finally, we compared somatic hypermutation levels in the V and J regions of each IgG subclass (IgG1–IgG4) between the three patients and nine healthy controls (Fig. 4D, left). Somatic hypermutation frequencies of IgG1, IgG2, and IgG3 were significantly lower in HSCT patients (*p* < 0.01) (Fig. 4D, left). Even in IgG4, although no superiority was observed, somatic hypermutation frequencies tended to be lower in HSCT patients (Fig. 4D, left). Control IgG consisted of sequences with the highest mutation, while IgM sequences showed almost no mutation. The distribution of mutations in HSCT patients showed greater mutation than in control IgM, but had lower levels of mutation than control IgG (Fig. 4D, right).

## Discussion

In this study, we used FACS, the QASAS method, and BCR repertoire analysis to assess the state of humoral immunity after HSCT. FACS analysis demonstrated that, in the early post-HSCT period, mature B cells were absent; instead, the compartment was dominated by highly immature cells that subsequently underwent gradual maturation. We interpret this change to represent the resetting of the immune system, resembling the establishment of a new immune system, similar to that of a newborn. Further, analysis using the QASAS method revealed that, despite antigen exposure prior to HSCT, the first vaccination after HSCT resulted in a primary immune pattern. We interpret this finding to mean that HSCT caused the loss of immune memory. Furthermore, when we compared the BCR repertoire of healthy controls and HSCT patients, we found that the repertoire of HSCT patients was more immature and closer to the initial state, which supports our interpretation.

B cells differentiate via a germinal center reaction, during which a high degree of somatic hypermutation is induced. IgG antibodies of healthy adults exhibited high somatic hypermutation levels and low pGen values, whereas post-transplant B cells showed low somatic hypermutation levels and high pGen values, a pattern characteristic of antigen-inexperienced B cells. These findings suggest that pre-transplant B-cell immune memory is lost following HSCT, and that the newly generated B cells remain functionally immature.

We propose that the response to vaccination after HSCT should be characterized by the fact that immune memory is reset by HSCT. To ensure the rapid acquisition of immunity, it may be appropriate to administer at least two doses of the primary vaccination series after HSCT. Tsoutsoukis et al. evaluated antibody responses following one to four doses of SARS-CoV-2 vaccine administered after HSCT and reported that antibody titers improved after the second and subsequent doses [18]. Furthermore, the U.S. guidelines recommend a three-dose regimen as the primary vaccination series after HSCT, highlighting the importance of multiple (i.e., two or more) vaccine doses in this population [19]. These reports support our suggestion.

Several limitations of our study warrant mention. First, immune reconstitution following HSCT is influenced by various distinct factors, and the results of this study may be affected by such variability. In our cohort, all three HSCT recipients underwent cord blood transplantation, and it is important to consider that immune reconstitution may differ when other hematopoietic stem cell sources are used. For example, previous studies have reported that the recovery of total T cells and natural killer cells after cord blood transplantation is comparable to that after bone marrow transplantation, whereas the reconstitution of CD4^+^ T cells and B cells tends to proceed more rapidly than in bone marrow transplantation or peripheral blood stem cell transplantation [20–24]. Therefore, further studies are needed to confirm whether similar results are observed in HSCT using other stem cell sources. In addition, factors such as the conditioning regimen and graft-versus-host disease prophylaxis may also influence immune reconstitution. Second, the impact of patient age on results should be considered. Previous reports have shown that the recovery of thymic function after HSCT affects immune reconstitution [4], and that the recovery of thymic function after HSCT is faster in children than in adults [25]. As our study included only three adult patients, the findings may not be generalizable to populations of different ages, including pediatric patients. Third, considering that some reports suggest immune reconstitution gradually matures over several years after transplantation [3, 23], the timing of vaccination after HSCT may affect the observed immune responses. In our study, the timing of vaccination varied substantially among patients (ranging from Day 392 to Day 656 post-transplantation), and we cannot rule out the possibility that this variability influenced the results. Taken together, these considerations highlight the need for further validation in diverse patient populations to generalize our findings.

Even with pre-HSCT exposure to antigen, first vaccination after HSCT induced a primary immune response. Regardless of past infection or vaccination history, the first vaccination after HSCT should be considered to induce a primary immune response.

## Supplementary Information

Below is the link to the electronic supplementary material.

Supplementary file1 (DOCX 30 KB)

## Data Availability

All data produced in the present study are available upon reasonable request to the authors.

## Declarations

### Clinical trial registration number

The study protocol was approved by Kobe University Hospital Ethics Committee (No. B2356701 and 1481).
