# Supplementary material for: Humoral immunity after hematopoietic stem cell transplantation: evaluation by B-cell receptor repertoire analysis
Source: Int J Hematol. 2025 Jul 25;122(6):877–84. doi: 10.1007/s12185-025-04042-9 (PMC12638405; doi:10.1007/s12185-025-04042-9)
Supplement: Supplementary file 1 — Supplementary file1 (DOCX 30 KB) [file 12185_2025_4042_MOESM1_ESM.docx]

Table S1

HSCT patients

|  | Age | Sex | Disease | Donor source | Conditioning regimen | GVHD prophylaxis |
| --- | --- | --- | --- | --- | --- | --- |
| Patient 1 | 53 | Female | Acute myeloid leukemia | Cord blood | Flu 25 mg/m^2^ × 5 days  MEL 40 mg/m^2^ × 2days  TBI 4 Gy/2 fr | TAC+MMF |
| Patient 2 | 58 | Male | Acute myeloid leukemia | Cord blood | Flu 25 mg/m^2^ × 5 days  MEL 40 mg/m^2^ × 2 days  TBI 4 Gy/2 fr | TAC+MMF |
| Patient 3 | 63 | Female | Acute myeloid leukemia | Unrelated bone marrow | Flu 30 mg/m^2^ × 6 days  Bu 3.2 mg/kg × 2 days  TBI 2 Gy/1 fr | TAC+MMF |
| Patient 4 | 48 | Male | B-lymphoblastic leukemia | Unrelated bone marrow | Flu 30 mg/m^2^ × 6 days  Bu 3.2 mg/kg × 2 days  TBI 2 Gy/1 fr | TAC+MMF |
| Patient 5 | 68 | Female | B-lymphoblastic leukemia | Unrelated bone marrow | Flu 30 mg/m^2^ × 6 days  Bu 3.2 mg/kg × 2 days  TBI 2 Gy/1 fr | TAC+MMF |
| Patient 6 | 64 | Male | Acute myeloid leukemia | Unrelated bone marrow | Flu 30 mg/m^2^ × 6 days  Bu 3.2 mg/kg × 2 days  TBI 2 Gy/1 fr | TAC+MMF |
| Patient 7 | 34 | Male | Mixed phenotype acute leukemia | Unrelated bone marrow | Flu 30 mg/m^2^ × 4 days  TBI 12 Gy/4 fr | TAC+MMF |
| Patient 8 | 22 | Male | Acute myeloid leukemia | Related bone marrow | Flu 30 mg/m^2^ × 4 days  TBI 12 Gy/4 fr | CyA+MMF |
| Patient 9 | 58 | Female | Myelodysplastic syndrome | Cord blood | Flu 25 mg/m^2^ × 5 days  MEL 40 mg/m^2^ × 2days  TBI 4 Gy/2 fr | TAC+MMF |
| Patient 10 | 37 | Female | Angioimmunoblastic T-cell lymphoma | Unrelated bone marrow | CY 60 mg/m^2^ × 2 days  TBI 12 Gy/4 fr | TAC+MMF |

Flu, fludarabine; MEL, melphalan; CY, cyclophosphamide; Bu, busulfan; TBI, total body irradiation; GVHD, graft versus host disease; TAC, tacrolimus; MMF, mycophenolate mofetil; CyA, cyclosporin

Table S2

Primary exposure

|  | **Individuals** | **Type of antigen exposure** | **Date of**  **QASAS analysis** |
| --- | --- | --- | --- |
| Case 1 | Infection patient 1 | Infection | Oct 2020 |
| Case 2 | Infection patient 2 | Infection | Sep 2020 |
| Case 3 | Infection patient 3 | Infection | Oct 2020 |
| Case 4 | Vaccine volunteer 1 | 1^st^ Vaccination (monovalent BNT162b2) | Apr 2021 |
| Case 5 | Vaccine volunteer 2 | 1^st^ Vaccination (monovalent BNT162b2) | May 2021 |
| Case 6 | Vaccine volunteer 3 | 1^st^ Vaccination (monovalent BNT162b2) | Apr 2021 |

Repeated exposure

|  | **Individuals** | **Type of antigen exposure** | **Date of**  **QASAS analysis** |
| --- | --- | --- | --- |
| Case 7 | Vaccine volunteer 1 | 2^nd^ Vaccination (monovalent BNT162b2) | Apr 2021 |
| Case 8 | Vaccine volunteer 1 | 5^th^ Vaccination (bivalent BNT162b2) | Nov 2022 |
| Case 9 | Vaccine volunteer 1 | 6^th^ Vaccination (monovalent XBB.1.5 BNT162b2) | Sep 2023 |
| Case 10 | Vaccine volunteer 2 | 6^th^ Vaccination (monovalent XBB.1.5 BNT162b2) | Sep 2023 |
| Case 11 | Vaccine volunteer 3 | 7^th^ Vaccination (monovalent mRNA-1273.815) | Oct 2023 |
| Case 12 | Vaccine volunteer 4 | 7^th^ Vaccination (DS-5670d) | Jan 2024 |
| Case 13 | Vaccine volunteer 5 | 5^th^ Vaccination (DS-5670d) | Feb 2024 |
| Case 14 | Vaccine volunteer 6 | 5^th^ Vaccination (DS-5670d) | Feb 2024 |

|  | **Age at HSCT** | **Type of antigen exposure** | **Date of**  **QASAS analysis** | **Days after HCST at time of vaccination** | **Date of final IVIG administration** | **Conditioning regimen** | **GVHD prophylaxis** |
| --- | --- | --- | --- | --- | --- | --- | --- |
| Patient 11 | 29 | 1^st^ Vaccination after cord blood transplantation (monovalent XBB.1.5 BNT162b2) | Dec/2023 | 420 | Dec 2022 | CY 60 mg/m^2^ × 2 days  TBI 12 Gy/4 fr | TAC+MMF |
| Patient 12 | 65 | 1^st^ Vaccination after cord blood transplantation (monovalent XBB.1.5 BNT162b2) | Nov/2023 | 392 | Jan 2023 | Flu 25 mg/m^2^ × 5 days  MEL 40 mg/m^2^ × 2 days  TBI 4 Gy/2 fr | TAC+MMF |
| Patient 13 | 63 | 4^th^ Vaccination after cord blood transplantation (monovalent XBB.1.5 BNT162b2) | Nov/2023 | 656 | Mar 2022 | Flu 25 mg/m^2^ × 5 days  MEL 40 mg/m^2^ × 2 days  TBI 4 Gy/2 fr | TAC+MMF |

Table S3

HSCT patients evaluated by the QASAS method

Flu, fludarabine, MEL, melphalan, CY, cyclophosphamide, TBI, total body irradiation, GVHD, graft versus host disease, TAC, tacrolimus, MMF, mycophenolate mofetil, IVIG, intravenous immunoglobulin
